# New Insights into FoxE1 Functions: Identification of Direct FoxE1 Targets in Thyroid Cells

**DOI:** 10.1371/journal.pone.0062849

**Published:** 2013-05-13

**Authors:** Lara P. Fernández, Arístides López-Márquez, Ángel M. Martínez, Gonzalo Gómez-López, Pilar Santisteban

**Affiliations:** 1 Instituto de Investigaciones Biomédicas “Alberto Sols”, Consejo Superior de Investigaciones Científicas, and Universidad Autónoma de Madrid (CSIC-UAM), Madrid, Spain; 2 Bioinformatics Unit, Structural Biology Program, Spanish National Cancer Research Centre (CNIO), Madrid, Spain; Consiglio Nazionale delle Ricerche (CNR), Italy

## Abstract

**Background:**

FoxE1 is a thyroid-specific forkhead transcription factor essential for thyroid gland development, as well as for the maintenance of the thyroid differentiated state in adults. FoxE1 recognizes and binds to a short DNA sequence present in thyroglobulin (*Tg*) and thyroperoxidase (*Tpo*) promoters, but FoxE1 binding to regulatory regions other than *Tg* and *Tpo* promoters remains almost unexplored. Improving knowledge of the regulatory functions of FoxE1 is necessary to clarify its role in endocrine syndromes and cancer susceptibility.

**Methodology/Principal Finding:**

In order to further investigate downstream FoxE1 targets, we performed a genome-wide expression screening after knocking-down *FoxE1* and obtained new insights into FoxE1 transcriptional networks in thyroid follicular cells. After validation, we confirmed *Adamts9*, *Cdh1*, *Duox2* and *S100a4* as upregulated genes and *Casp4*, *Creld2*, *Dusp5*, *Etv5*, *Hsp5a*, *Nr4a2* and *Tm4sf*1 as downregulated genes when *FoxE1* was silenced. In promoter regions of putative FoxE1-regulated genes and also in the promoters of the classical thyroid genes *Nis*, *Pax8* and *Titf1*, we performed an *in silico* search of the FoxE1 binding motif that was in close proximity to the NF1/CTF binding sequence, as previously described for other forkhead factors. Using chromatin immunoprecipitation we detected specific *in vivo* FoxE1 binding to novel regulatory regions in two relevant thyroid genes, *Nis* and *Duox2.* Moreover, we demonstrated simultaneous binding of FoxE1 and NF1/CTF to the *Nis* upstream enhancer region, as well as a clear functional activation of the Nis promoter by both transcription factors.

**Conclusions/Significance:**

In search for potential downstream mediators of FoxE1 function in thyroid cells, we identified two novel direct FoxE1 target genes. To our knowledge, this is the first evidence regarding the implication of *Nis* and *Duox2* in executing the transcriptional program triggered by FoxE1. Furthermore, this study points out the important role of FoxE1 in the regulation of a large number of genes in thyroid cells.

## Introduction

Coordinated expression of thyroid transcription factors Pax8, FoxE1/Ttf2 and Ttf1/Nkx2-1 is essential for maintaining the differentiated thyroid function, which involves synthesis and secretion of thyroid hormones. These factors are encoded by genes with paired box, forkhead box and homeobox domains, respectively. Thyroid hormones are iodinated, and therefore thyroid cells actively concentrate iodide through a sodium dependent co-transporter, Nis, a glycoprotein located in the basal membrane. The iodide is transported to the apical membrane, where thyroperoxidase (Tpo) iodinates the tyrosine residues of the main thyroid protein thyroglobulin (Tg) that serves as a storage for thyroid hormones [1], [2].

FoxE1, formerly known as thyroid transcription factor 2 or Ttf2, is a thyroid-specific transcription factor that belongs to the forkhead/winged-helix family [3]. Fox proteins are a superfamily of evolutionarily conserved transcriptional regulators, which share a highly conserved forkhead box or winged helix DNA binding domain. Forkhead factors control a wide range of biological processes, and some of them are key regulators of embryogenesis and play important roles in cell differentiation and development, hormone responsiveness and aging [4], [5].

FoxE1, as a member of the Fox family, is able to interact with nucleosomes through its winged-helix DNA binding domain and to alter chromatin structure, creating a locally exposed domain necessary for the action of other transcription factors [6]. This intrinsic property defines FoxE1 as a pioneer transcription factor [7], essential during thyroid development and differentiation, as well as for the maintenance of the thyroid differentiated state in adults [2].

*Foxe1*-null mice exhibit an ectopic (50%) or completely absent (50%) thyroid gland and severe cleft palate. Consequently, FoxE1 seems to be crucial either for the migration of the thyroid cell precursors or for repressing differentiation until migration has completed [8].

Human *FOXE1* mutations cause the Bamforth–Lazarus syndrome (OMIM 241850), which is associated with congenital hypothyroidism, cleft palate and spiky hair, with or without choanal atresia, bifid epiglottis and ocular hypertelorism [9], [10]. Moreover, *FOXE1* variations have been associated with susceptibility to several types of cancer [11], [12], [13], including papillary thyroid cancer [14], [15], [16].

FoxE1 was initially identified as a nuclear protein [3] that recognizes and binds to DNA sequences present in the promoters of two thyroid-specific genes: thyroglobulin *(Tg*) [17] and thyroperoxidase *(Tpo)* [18]. FoxE1 usually promotes transcriptional activation of the *Tg* and *Tpo* genes; however, it can also act as a promoter-specific transcriptional repressor of both genes [19].

Putative FoxE1-binding sites previously identified in the *Tg* and *Tpo* promoters share the core sequence AAACA [20]. Moreover, in the *Tpo* promoter FoxE1 forms part of an interaction-complex together with the transcription factor NF1/CTF, whose final result is to turn on the expression of the *Tpo* gene in response to external hormonal stimuli [21].

Nevertheless, FoxE1 binding to DNA sequences other than the *Tg* and *Tpo* promoters remains almost unexplored. Only two studies have reported other FoxE1 targets, but both were conducted in heterologous expression systems [22], [23].

In order to further investigate FoxE1 downstream targets in thyroid epithelial cells, we performed a genome-wide screening using expression arrays in *FoxE1* knock-down cells followed by a search of direct target genes containing in their promoters both FoxE1 and NF1/CTF binding sites. The results obtained in this study provide new insights into FoxE1 transcriptional networks in differentiated thyroid cells and predict involvement of FoxE1 in relevant biological processes and pathways. These data may lead to a better understanding of thyroid biology.

## Materials and Methods

### Cell Culture

PCCl3 cells, a continuous line of rat thyroid follicular cells [24], were cultured in Coon’s modified Ham’s F-12 medium supplemented with 5% donor calf serum and a six-hormone mixture [25]. These cells express thyroid-specific genes (*Tg*, *Tpo*, and *Nis*) as well as thyroid-specific transcription factors (Ttf1, FoxE1, and Pax8) [26] and therefore constitute a good model system to study differentiation and growth regulation in a thyroid epithelial cell setting.

HeLa cells were grown in Dulbecco’s modified Eagle’s medium (DMEM) supplemented with 5% fetal bovine serum.

### RNA Interference

PCCl3 cells were transfected with 25 nM of FoxE1 siRNA (Rat FoxE1 ON-TARGETplus SMARTpool) or with scrambled siRNA (ON-TARGETplus Non-targeting Pool) using DharmaFECT 1 Transfection Reagent and following the manufacturer’s protocol (Dharmacon, Denver, USA).

In order to establish optimal conditions for *FoxE1* silencing, duplicate samples were harvested at different time points (24, 48 and 72 h) after transfection, and total protein was extracted. *FoxE1* silencing was tested by western blotting using a polyclonal FoxE1 antibody (Biopat, Milan, Italy) (data not shown). Once 48 hours was defined as the best time point for *FoxE1* silencing, we performed additional transfections, four biological replicates for each condition, to isolate total protein and total RNA using TRIzol reagent (Invitrogen, Carlsbad, CA) following the manufacturer’s recommended protocol.

### Expression Arrays

**Figure 1:**
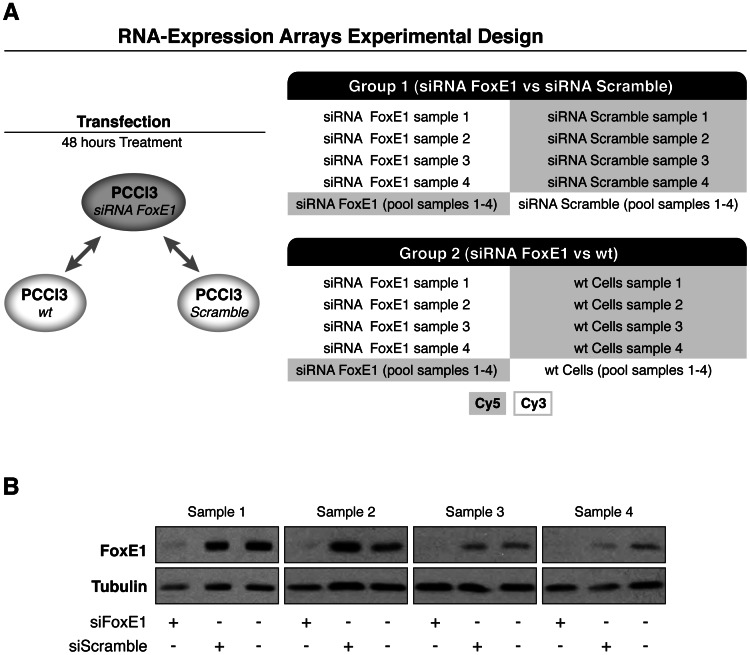
Experimental design and FoxE1 protein levels after 48 hours of silencing. *Panel A:* Experimental design of expression array experiments. Two comparisons were done: FoxE1-silenced PCCl3 cells (siFoxE1 PCCl3) *vs.* scrambled siRNA-treated PCCl3 cells (siScramble PCCl3), and FoxE1-silenced PCCl3 cells *vs.* wild type PCCL3 cells (wt PCCl3). Each comparison was performed in quadruplicate and using dye swaps. *Panel B:* Western Blot of extracts from control, siScramble and siFoxE1-treated cells from quadruplicate samples used for microarray analysis. Hybridizations were done with anti-FoxE1 antibodies; anti-tubulin antibodies were used as loading controls.

FoxE1-dependent gene expression was tested using expression arrays (Agilent SurePrint Rat 60 K). We established two main comparisons: FoxE1-silenced PCCl3 cells (siFoxE1 PCCl3) vs. scrambled siRNA-treated PCCl3 cells (siScramble PCCl3) and FoxE1-silenced PCCl3 cells vs. wild type PCCL3 cells (wt PCCl3). This last condition was included to consistently analyse expression array signals in basal cellular conditions. Each comparison was performed using quadruplicates and dye swaps (Experimental design shown in Fig. 1A).

Ten µg of total RNA for each condition were sent to the Genomics Core Unit of the Spanish National Cancer Research Centre (CNIO, Madrid) for RNA quality evaluation, amplification, labelling and hybridization to Agilent SurePrint Rat 60 K arrays according to the manufacturer’s protocols.

Signal quantification was carried out with Agilent Feature Extraction Software 10.7 (Agilent Technologies, Palo Alto, CA), using default analysis parameters for Agilent’s whole rat genome 60 K gene expression arrays. To normalize the data set, we performed loess within-array normalization and quantiles between-array normalization. Differential expression analysis was done using Bioconductor’s limma package (http://www.bioconductor.org). At a later stage, we used the file “SurePrint G3 Rat GE 8×60 K Microarray” to obtain the annotations of the rat genome from Agilent. Genes that showed adjusted p-values <0.005 both in siFoxE1 *vs*. wild type and in siFoxE1 *vs.* siScramble PCCl3 cells were considered differentially expressed. Functional analysis of Gene Ontology (GO) terms was carried out using the FatiGO tool and gene set enrichment analysis was performed using FatiScan [27], [28]. All microarray data can be downloaded from the Gene Expression Omnibus (GEO; http://www.ncbi.nlm.nih.gov/geo/) database under accession number GSE42497.

### Experimental Validation of Expression Array Data

Technical validations of expression data were performed by real-time quantitative PCR (qPCR) and western blots after performing four independent experiments of *FoxE1* silencing.

Total RNA was isolated from PCCl3 cells after silencing following the standard TRIzol Reagent protocol (Invitrogen, Carlsbad, CA). RNA was reverse transcribed with random primers and quantitative PCR reactions were assembled in triplicate using the SYBR Green PCR Kit (Kapa Biosystems, Woburn, MA) following the manufacturer’s protocol. Expression level changes were studied for 6 thyroid-specific genes (*FoxE1, Tpo, Tg, Nis, Pax8* and *Titf1*) as well as for 12 putative FoxE1-regulated genes, using beta-glucuronidase (*Gus*) for gene expression normalization (oligos are shown in Table S1).

Total protein extracts were obtained by scraping the cells in RIPA buffer (1% PBS, 0.5% (wt/vol) Nonidet, 0.1% (wt/vol) sodium deoxycholate, 0.1% SDS). Protein extracts (30 µg) were separated on 10% SDS-PAGE gels and immunodetected after western blotting. FoxE1 antibody was from Biopat (Milan, Italy), Actin and Cdh1 antibodies were from Santa Cruz Biotechnology, Inc. (Santa Cruz, CA), and Nis and Duox2 antibodies were generous gifts from Dr. N. Carrasco (Department of Cellular and Molecular Physiology, Yale School of Medicine, New Haven, CT, USA) [29] and Dr. C. Dupuy (Institut Gustave Roussy,Villejuif, France) [30], respectively.

### Motif Search

We searched for FoxE1 binding sites in promoter regions (+/−1000 bp relative to the transcription start site) of genes statistically significantly regulated by FoxE1 (p-values <0.005). We performed an automated search of genes containing both the FoxE1 core binding site [5′-ACAAA-3′] and the NF1/CTF binding motif [5′-TTGG -3′] localised between 5 to 30 bp from the FoxE1 site, as previously described for FoxE1 binding within the *Tpo* promoter [5′-TTGG-(5–30)bp-ACAAA-3′] [21]. FASTA promoter sequences of FoxE1-regulated genes, as well as promoter sequences of their orthologous genes, were extracted from the Ensembl database (http://www.ensembl.org).

### ChIP and ReChIP Assays

Chromatin immunoprecipitation (ChIP) and sequential ChIP (ReChIP) were performed using the Diagenode HighCell ChIP kit following the manufacturer’s standard protocol. Cross-linked PCCl3 chromatin was immunoprecipitated using polyclonal antibodies against FoxE1 (Biopat, Milan, Italy) and NF1 (Abcam, Cambridge, UK). Two independent ChIP experiments were performed using two different batches of Biopat FoxE1 antibodies.

ReChIP was used to analyse simultaneous binding of FoxE1 and NF1/CTF proteins to a common DNA region. After a first immunoprecipitation with FoxE1 antibody using the Diagenode HighCell ChIP kit, samples were rinsed with RIPA buffer, and the first antibody was stripped from the beads by incubating in 1% SDS at 65°C for 15 minutes. Then a second round of immunoprecipitation with NF1 antibody was performed using the same Diagenode HighCell ChIP kit and following the manufacturer’s standard protocol.

FoxE, NF1 and FoxE1/NF1 -immunoprecipitated and input samples were analysed by real-time PCR using specific primers for the analysed regions (see Table S2). The known FoxE1 targets *Tpo* and *Tg* were used as positive IP controls whereas intron and promoter regions of *Gad1* (glutamate decarboxylase 1) and *Afm* (afamin or alpha-albumin) were used as negative controls. qPCR reactions were assembled in triplicate using the SYBR Green PCR Kit (Kapa Biosystems, Woburn, MA), following the manufacturer’s protocol. The enrichment of target sequences in ChIP material was calculated relative to the *Afm* and *Gad1* negative controls, and normalized to their relative amplification in the input sample [31].

### Transfection and Luciferase Assays

HeLa cells were plated at a density of 2×10^5^ cells/well in 6-well tissue culture plates, 24 h before transfection. Transfections were performed by calcium phosphate co-precipitation as described previously [17]. The rat promoter pNIS 2.8-Luc, which contains the NIS Upstream Enhancer (NUE) [32] was transiently transfected alone (1.5 µg) or in combination with 1 µg of the expression vectors CMV-FoxE1 (formerly CMV-TTF2) [3] or RSV-CTF/NF1-C [33]. One hundred nanograms of the CMV-*Renilla* vector were cotransfected to assess transfection efficiency. After 48 h, cells were harvested, lysed, and analysed for Luciferase and Renilla activities by the Dual-Luciferase reporter assay system (Promega, Madison WI). The promoter activity in cells transfected with expression vectors was determined as the ratio between luciferase and Renilla activities, relative to the ratio obtained in cells transfected with the corresponding control vectors. The results shown are the average ± SD of six different experiments performed in triplicate. Statistical significance was determined by *t* test analysis (two-tailed), and differences were considered significant at *p*<0.05. Western blots were done where indicated to determine the levels of expression of the transfected expression vectors (data not shown).

## Results

### Gene Expression Profile of FoxE1-silenced Cells

In order to further investigate FoxE1 function and to identify putative FoxE1 target genes in thyroid cells, we performed whole genome microarray analysis in PCCl3 thyroid cells after knocking down *FoxE1*. Silencing was performed for 48 h after transfection. Data from four independent silencing experiments were analysed. We obtained a FoxE1 silencing grade of more than 85% (Fig. 1B). Statistically significant probes (p<0.005) from the siFoxE1 PCCl3 *vs* siScrambl PCCl3 comparison showed 74 differentially deregulated probes, including 24 upregulated and 50 downregulated probes. Comparison of siFoxE1 PCCl3 *vs* wt PCCl3 generated 211 differentially regulated probes, consisting of 99 upregulated and 112 downregulated probes (Table 1). Array results of both comparisons are shown in datasets S1 and S2, respectively. Statistically significant probes (p<0.005) from both comparisons are shown in Table S3. Combination of significant results from both comparisons showed 55 differentially deregulated genes, 17 of which were upregulated and 38 were downregulated (Tables 1 and 2). As expected, expression of previously known FoxE1 targets (*Tpo* and *Tg*) was downregulated, in the absence of FoxE1, in both comparisons.

**Table 1:** Summary of FoxE1 microarray results in PCCl3 cells.

	siFoxE1 *vs* siScrambl		siFoxE1 *vs* WT		Common
p-value<0.005	Probes	Genes	Probes	Genes	Genes
Total	74	64	211	183	55
Upregulated	24	24	99	87	17
Downregulated	50	40	112	96	38

Number of statistically significant probes and genes (p<0.005) after Foxe1 silencing; results are shown for each type of comparison performed, as well as for the combined analysis.

**Table 2:** Description of statistically genes significantly (p<0.005) regulated by FoxE1.

Gene	Status	Selected for validation	Description
***Ahcy***	Downreg.	No	Catalyzes the reversible hydrolysis of S-adenosylhomocysteine (AdoHcy) to adenosine (Ado) and L-homocysteine (Hcy).
***Amigo3***	Downreg.	No	Adhesion molecule with Ig-like domain 3.
***Ankrd37***	Downreg.	No	Ankyrin repeat domain 37, ANKRD37 is a novel HIF-1-target gene.
***Atmin***	Downreg.	No	ATM interactor, an essential cofactor for checkpoint kinase ATM, ATMIN.
***Bet1***	Downreg.	No	This gene encodes a golgi-associated membrane protein that participates in vesicular transport from the endoplasmic reticulum (ER) to the Golgi complex.
***Casp4***	Downreg.	Yes	Caspase 4, apoptosis-related cysteine peptidase; sequential activation of caspases plays a central role in the execution phase of cell apoptosis.
***Coq10b***	Downreg.	No	Coenzyme Q10 homolog B (S. cerevisiae).
***Creld2***	Downreg.	Yes	Cysteine-rich with EGF-like domains 2; the CRELD family has widely diverse biological roles in both developmental events and subsequent cell function.
***Ctgf***	Downreg.	No	The encoded protein plays a role in chondrocyte proliferation and differentiation, cell adhesion in many cell types, and is related to platelet-derived growth factor.
***Ddit3***	Downreg.	No	DNA-damage-inducible transcript 3; the protein is implicated in adipogenesis and erythropoiesis, is activated by endoplasmic reticulum stress, and promotes apoptosis.
***Derl3***	Downreg.	No	Der1-like domain family, member 3: this protein appears to be involved in the degradation of misfolded glycoproteins in the ER.
***Dnajb11***	Downreg.	No	DNAJB11 belongs to the evolutionarily conserved DNAJ/HSP40 family of proteins, which regulate molecular chaperone activity by stimulating ATPase activity.
***Dnajb9***	Downreg.	No	DnaJ (Hsp40) homolog, subfamily B, member 9; this protein is induced by endoplasmic reticulum stress and plays a role in protecting stressed cells from apoptosis.
***Dnajc3***	Downreg.	No	It is a member of the tetratricopeptide repeat family of proteins and acts as an inhibitor of the interferon-induced, dsRNA-activated protein kinase (PKR).
***Dusp5***	Downreg.	Yes	It negatively regulates members of the mitogen-activated protein (MAP) kinase superfamily (MAPK/ERK, SAPK/JNK, p38), which are associated with cellular proliferation and differentiation.
***Engase***	Downreg.	No	Endo-beta-N-acetylglucosaminidase, (ENGase; EC 3.2.1.96) is involved in the processing of free oligosaccharides in the cytosol.
***Ero1lb***	Downreg.	No	ERO1-like beta (S. cerevisiae), endoplasmic oxidoreductin-1-like protein B.
***Etv5***	Downreg.	Yes	Ets variant gene 5; ETV5 belongs to the PEA3 subfamily of Ets transcription factors.
***Ggct***	Downreg.	No	Gamma-glutamylcyclotransferase; the encoded protein may also play a role in cell proliferation, and the expression of this gene is a potential marker for cancer.
***Gmppb***	Downreg.	No	GDP-mannose pyrophosphorylase B; catalyzes the conversion of mannose-1-phosphate and GTP to GDP-mannose, a reaction involved in the production of N-linked oligosaccharides.
***Hsp90b1***	Downreg.	No	Heat shock protein 90 kDa beta (Grp94), member 1; HSP90 proteins have key roles in signal transduction, protein folding, protein degradation, and morphologic evolution.
***Hspa5***	Downreg.	Yes	Heat shock 70 kDa protein 5 (glucose-regulated protein, 78 kDa); as this protein interacts with many ER proteins, it may play a key role in monitoring protein transport through the cell.
***Hyou1***	Downreg.	No	Hypoxia upregulated 1; the protein encoded by this gene belongs to the heat shock protein 70 family. This gene uses alternative transcription start sites.
***Igf2bp2***	Downreg.	No	Insulin-like growth factor 2 mRNA binding protein 2; it functions by binding to the 5′ UTR of the insulin-like growth factor 2 (IGF2) mRNA and regulating IGF2 translation.
***Il23a***	Downreg.	No	Interleukin 23, alpha subunit p19; both IL23 and IL12 can activate the transcription activator STAT4, and stimulate the production of interferon-gamma (IFNG).
***LOC365444***	Downreg.	No	Similar to CGI-09 protein.
***Manf***	Downreg.	No	Mesencephalic astrocyte-derived neurotrophic factor. Reducing expression of this gene increases susceptibility to ER stress-induced death and promotes cell proliferation.
***Mfsd2***	Downreg.	No	Major facilitator superfamily domain containing 2A. Mfsd2a plays a role in adaptive thermogenesis, it is a lung tumor suppressor gene that regulates cell cycle progression and matrix attachment.
***Nr4a2***	Downreg.	Yes	Nuclear receptor subfamily 4, group A, member 2; this gene encodes a member of the steroid-thyroid hormone-retinoid receptor superfamily. The encoded protein may act as a transcription factor.
***Nupr1***	Downreg.	No	Nuclear protein, transcriptional regulator 1. It was originally identified as p8, a member of the family of HMG-I/Y transcription factors induced in response to various cellular stressors.
***Pdia4***	Downreg.	No	Protein disulfide isomerase family A, member 4.
***Riok3***	Downreg.	No	RIO kinase 3 (yeast); the specific function of this gene has not yet been determined.
***Sdf2l1***	Downreg.	No	Stromal cell-derived factor 2-like 1, a component of the endoplasmic reticulum chaperone complex.
***Sec23b***	Downreg.	No	Sec23 homolog B (S. cerevisiae). The protein encoded by this gene is a member of the SEC23 subfamily of the SEC23/SEC24 family, which is involved in vesicle trafficking
***Sel1l***	Downreg.	No	Sel-1 suppressor of lin-12-like is part of a protein complex required for the retrotranslocation or dislocation of misfolded proteins.
***Tm4sf1***	Downreg.	Yes	Transmembrane 4 L six family member 1. The proteins mediate signal transduction events that play a role in the regulation of cell development, activation, growth and motility.
***Tmem66***	Downreg.	No	Transmembrane protein 66.
***Zfand2a***	Downreg.	No	Zinc finger, AN1-type domain 2A.
***Adamts9***	Upreg.	Yes	ADAM metallopeptidase with thrombospondin type 1 motif, 9. Members of the ADAMTS family have been implicated in the cleavage of proteoglycans, organ development, and angiogenesis.
***Bcam***	Upreg.	No	Basal cell adhesion molecule, member of the immunoglobulin superfamily and a receptor for the extracellular matrix protein laminin.
***Cdh1***	Upreg.	Yes	Cadherin 1; the encoded protein is a calcium dependent cell-cell adhesion glycoprotein.
***Crip2***	Upreg.	Yes	Cysteine-rich intestinal protein 2 (CRIP2); acts as a repressor of NF-kappaB-mediated proangiogenic cytokine transcription to suppress tumorigenesis and angiogenesis.
***Duox2***	Upreg.	Yes	It is a glycoprotein and a member of the NADPH oxidase family; the synthesis of thyroid hormone is catalyzed by a protein complex located at the apical membrane of thyroid follicular cells.
***Dynlrb2***	Upreg.	No	Dynein light chain roadblock-type 2
***Elovl2***	Upreg.	No	ELOVL fatty acid elongase 2.
***Fgf18***	Upreg.	No	Fibroblast growth factor 18; involved in a variety of biological processes, including embryonic development, cell growth, morphogenesis, tissue repair, tumor growth, and invasion.
***Folr1***	Upreg.	No	This gene product is a secreted protein that either anchors to membranes via a glycosyl-phosphatidylinositol linkage or exists in a soluble form.
***Krt20***	Upreg.	No	Keratin 20; the keratins are intermediate filament proteins responsible for the structural integrity of epithelial cells and are subdivided into cytokeratins and hair keratins.
***Prima1***	Upreg.	No	Proline-rich membrane anchor 1; the product of this gene functions to organize acetylcholinesterase (AChE) into tetramers, and to anchor AChE at neural cell membranes.
***Prss8***	Upreg.	No	Protease, serine, 8; this gene encodes a trypsinogen, which is a member of the trypsin family of serine proteases.
***Ril***	Upreg.	No	Reversion-induced LIM gene, binds alpha-actinin; may regulate actin stress fiber turnover.
***RT1-Da***	Upreg.	No	MHC class II RT1D alpha chain antigen.
***S100a4***	Upreg.	Yes	S100 calcium binding protein A4; S100 proteins are involved in the regulation of a number of cellular processes such as cell cycle progression and differentiation.
***Slit1***	Upreg.	No	Slit homolog 1; the genes encoding Slits and their Robo receptors are silenced in many types of cancer, including breast, suggesting a role for this signaling pathway in suppressing tumorigenesis.
***Tmem140***	Upreg.	No	Transmembrane protein 140.

Gene ontology (GO) analysis using FatiGO identified common GO terms, including endoplasmic reticulum (ER) overload response (GO:0006983), response to ER stress (GO:0034976), cellular response to glucose starvation (GO:0042149), protein folding (GO:0006457), heat shock protein binding, and oxidoreductase activity acting on sulfur group of donors, disulfide as acceptor (GO:0016671).

### Independent Validation of Genes Regulated by FoxE1 Silencing

**Figure 2:**
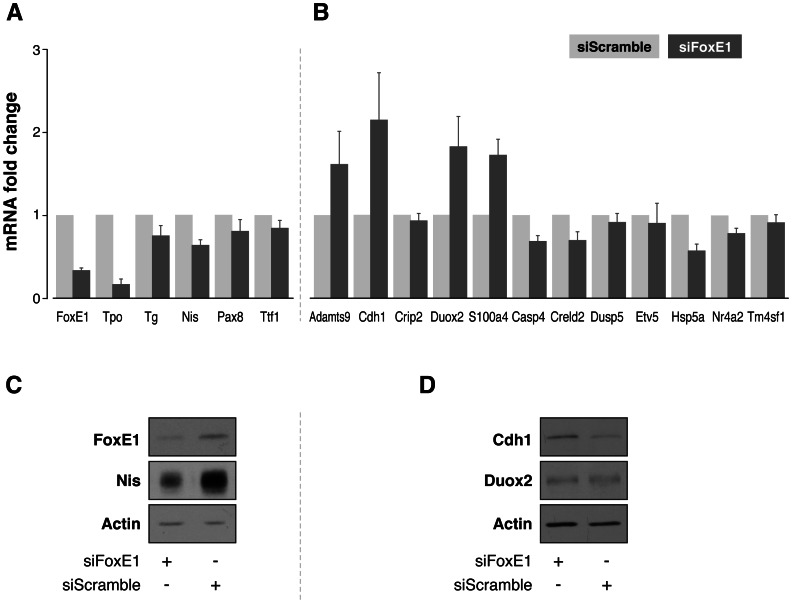
Experimental validation of microarray results by qRT-PCR and western blotting. Relative expression assessed by means of qRT-PCR of 6 thyroid-specific genes (panel A) and 12 additional genes differentially down- and upregulated in FoxE1-silenced PCCl3 cells (panel B). As FoxE1-dependent positive controls, we evaluated Tpo and Tg mRNA expression levels. Relative gene expression in siFoxE1 samples was calculated using the corresponding siScrambl samples as a reference ( = 1). Results are mean ± SEM of four independent experiments. Total protein extracts were prepared and submitted to western blot analysis to assess the protein levels of FoxE1 and Nis (panel C), and of Cdh1 and Duox2 (panel D). Actin was used as loading control. Representative western blot assays of four independent experiments are shown.

We performed validations of microarray data using four independent *FoxE1* silencing experiments, 48 h after siRNA transfection. First of all, we analysed expression levels of FoxE1, in order to check and confirm its inhibition at the mRNA level. We also checked the mRNA expression status of the FoxE1 target genes *Tpo* and *Tg*, as well as mRNA levels of the classical, most representative, thyroid genes: the thyroid iodide symporter gene, *Nis,* and thyroid transcription factors *Pax8* and *Ttf1* (Fig. 2A).

Moreover, we validated 12 genes randomly chosen from the list of putative FoxE1 targets, 5 of which were upregulated and 7 of which were downregulated after *FoxE1* silencing (Table 2). Genes selected for this validation included: metallopeptidase *Adamts9*; cadherin 1 (*Cdh1*), a cell-cell adhesion glycoprotein; *Crip2*, a repressor of NF-kappaB; dual oxidase 2 (*Duox2*), a glycoprotein located at the apical membrane of thyroid follicular cells; *S100a4*, a calcium-binding protein; caspase 4 (*Casp4*), an apoptosis-related cysteine peptidase; the cysteine-rich with GF-like domains protein 2, *Creld2*; dual specificity phosphatase 5 (*Dusp5*); transcription factor *Etv5*; 70 kDa heat shock protein 5 (*Hsp5a*); steroid-thyroid hormone-retinoid receptor *Nr4a2*; and transmembrane 4L six family member 1, *Tm4sf1*.

After *FoxE1* silencing, qRT-PCR analysis confirmed downregulation of FoxE1 as well as its previously described target genes *Tpo* and *Tg* [17], [18]. Interestingly, we also observed inhibition of *Nis*, and its mRNA levels were decreased even more than *Tg* mRNA levels (Fig. 2A). We confirmed the microarray data for selected genes (Fig. 2B) with the only exception of the *Crip2* gene, whose qRT-PCR expression levels did not show differences after *FoxE1* silencing. All validation experiments were performed four times, attesting to the robustness of the results.

We confirmed that mRNA levels of *Adamts9*, *Cdh1*, *Duox2* and *S100a4* were upregulated, and those of *Casp4*, *Creld2*, *Dusp5*, *Etv5*, *Hsp5a*, *Nr4a2* and *Tm4sf1* were downregulated when FoxE1 was silenced in thyroid cells.

The protein expression of FoxE1 was analysed in order to check and confirm its inhibition at the protein level. We then checked protein levels of three FoxE1 putative target genes, *Nis*, *Duox2* and *Cdh1* (Figs 2C and 2D). After *FoxE1* silencing, Duox2 protein levels did not change, but we confirmed inhibition of Nis expression and overexpression of Cdh1, at protein level.

### Identification and Functional Analysis of Direct FoxE1 Targets

With the aim of investigating new direct FoxE1 targets, first we searched for the specific FoxE1 core binding sequence, AAACA, in promoter regions of putative FoxE1 regulated genes (+/−1000 pb). However, AAACA is a short sequence that can be easily found at random in the genome. We therefore performed an automatic search in the promoter sequences for FoxE1 binding sites in close proximity (5–30 bp) to the NF1/CTF binding motif, [5′-TTGG-(5–30)bp-ACAAA-3′], as previously described for FoxE1 binding to the *Tpo* promoter [21] and for other forkhead family members [34].

FoxE1-NF1/CTF binding sequences were present in promoter regions of 26 out of 55 differentially FoxE1-regulated genes. Results are summarized in Table 3. Considering their previously described function in thyroid biology, we selected three of these genes: *Duox2*, *Cdh1* and *Nr4a2* for performing ChIP *in vivo* analysis of FoxE1 binding to their promoters.

**Table 3:** Summary of FoxE1- NF1/CTF binding motif search.

Gene	Status	X bp	Strand	Sequence	% Sequence in orthologous	Average X bp in orthologous	Min Xbp	Max Xbp
***Tm4sf1***	Downreg.	29	−1	GGA**TGTTT**-CTCACATCCTACTACAGGGTTTCGTTGTC-**CCAA**GGA	70.6	23.5	6	29
***Etv5***	Downreg.	29	1	TGA**TTGG**-TCAATGGCGCCCAACGCATAAATTATGTA-**AAACA**GAC	50.0	20.3	11	29
***Hspa5***	Downreg.	10	1	GAC**TTGG**-CAGAAAAGAA-**AAACA**CTG	41.8	11.7	8	20
***Casp4***	Downreg.	8	−1	TTG**TGTTT**-GCTTGTTC-**CCAA**GAG	41.4	20.3	8	29
***RT1-Da***	Upreg.	11	−1	GTG**TGTTT**-TACCAACATTT-**CCAA**ACT	39.7	19.4	5	30
***Sel1l***	Downreg.	11	1	GGT**TTGG**-GAGAGCTGTTG-**AAACA**TTT	38.6	13.6	5	29
***Ero1lb***	Downreg.	18	−1	GAG**TGTTT**-TCACTACATGCTAGAAAG-CCAAGGC	35.6	18.2	6	29
***Elovl2***	Upreg.	26	−1	GTC**TGTTT**-AGACTGAATATTAGTGCTCACATGCT-**CCAA**GTG	35.4	15.1	7	26
***Nr4a2***	Downreg.	22	1	CTT**TTGG**-AATATCCGGGAGTGTAGACCCT-**AAACA**GCT	31.6	22.8	6	30
***Dnajb9***	Downreg.	18	1	TTT**TTGG**-CCTCAGTTTTCGTAGGAG-**AAACA**GGG	31.5	16.6	5	29
***Manf***	Downreg.	24	1	AGA**TTGG**-TTCGTCAATTCACGTGGTAGTTCA-**AAACA**AGA	31.4	15.9	5	30
***RT1-Da***	Upreg.	29	1	GCT**TTGG**-GGCTCAGTTTATTTTCCAGTGTCCTCAGA-**AAACA**GCA	29.3	17.5	7	30
***Bet1***	Downreg.	16	1	TAA**TTGG**-ACCCCATGCTCCACCC-**AAACA**AGT	28.1	15.4	5	30
***Dynlrb2***	Upreg.	12	1	GGG**TTGG**-GGGGTGAGCAAC-**AAACA**AAG	27.5	19.6	9	26
***Duox2***	Upreg.	26	1	TTC**TTGG**-AGCCCCGGGCAGGGAGCGGAGAAGAG-**AAACA**AAA	26.6	19.8	7	28
***Crip***	Upreg.	27	1	GCC**TTGG**-CACCCCTCCCATGACCATGGCAACAGG-**AAACA**ACA	25.7	22.7	6	30
***Hsp90b1***	Downreg.	30	−1	CCC**TGTTT**-GCATTCCGACCTGTGATAGTTAAGCGAGGC-**CCAA**GTT	25.4	22.1	7	30
***Dnajc3***	Downreg.	21	1	ATC**TTGG**-GTGTCAGAGCGGGGAGGAGTT-**AAACA**TGG	24.1	19.6	7	29
***Sec23b***	Downreg.	6	1	TTT**TTGG**-ATGTCT-**AAACA**TGT	23.6	17.4	6	28
***Engase***	Downreg.	24	1	TGC**TTGG**-GGCCTCATAAAGCTCAAAGCCTGT-**AAACA**GTA	22.9	16.4	5	29
***Cdh1***	Upreg.	10	1	TAC**TTGG**-GCTGGAAAAC-**AAACA**AAA	22.2	13.4	5	29
***Slit1***	Upreg.	28	−1	GAG**TGTTT**-CTTAATTTTTCATCTATAACTACTTAGC-**CCAA**CTA	21.4	18.6	9	28
***Tmem140***	Upreg.	23	−1	TAG**TGTTT**-CTGGGAGCTGTCTACAGTCTTTC-**CCAA**GCT	21.1	15.8	6	26
***Creld2***	Downreg.	28	−1	AAG**TGTTT**-ATCTGATGTAATGAGGCCTACTTGTAAA-**CCAA**AGA	18.5	21.6	10	30
***Duox2***	Upreg.	27	−1	CGG**TGTTT**-ATCAGGCTCTGCAGGAGCCGCTACTCC-**CCAA**CGC	17.9	22.9	12	28
***Hyou1***	Downreg.	23	−1	AGG**TGTTT**-GACAGGTACAGTGCTGTGGTGTG-**CCAA**TTA	17.9	16.4	5	28
***Ctgf***	Downreg.	11	1	TTC**TTGG**-TGTTGTGCTGG-**AAACA**CAA	16.7	14.3	5	27
***Slit1***	Upreg.	23	1	ATC**TTGG**-AGGGTCATGATGGGGATGATAGG-**AAACA**AAG	15.9	15.9	5	26
***Tmem140***	Upreg.	29	1	GCT**TTGG**-GACACATTGTTCTTCAGAAGGAGGCAGAC-**AAACA**AAA	15.6	23.5	11	29
***Ankrd37***	Downreg.	30	1	CAG**TTGG**-TATAAAGAGCCATGTAAGAATTTCCAGGAA-**AAACA**TAC	15.0	21.3	7	30

Genes statistically significantly regulated by FoxE1, showing FoxE1-NF1/CTF binding motifs in their promoter regions (+/−1000 pb). Status of their expression in the absence of FoxE1, sequence in their promoter regions containing the FoxE1-NF1/CTF motif, and base pair distance between NF1/CTF and FoxE1 motifs are detailed. Information regarding the presence of these motifs in their orthologous sequence is shown.

**Figure 3:**
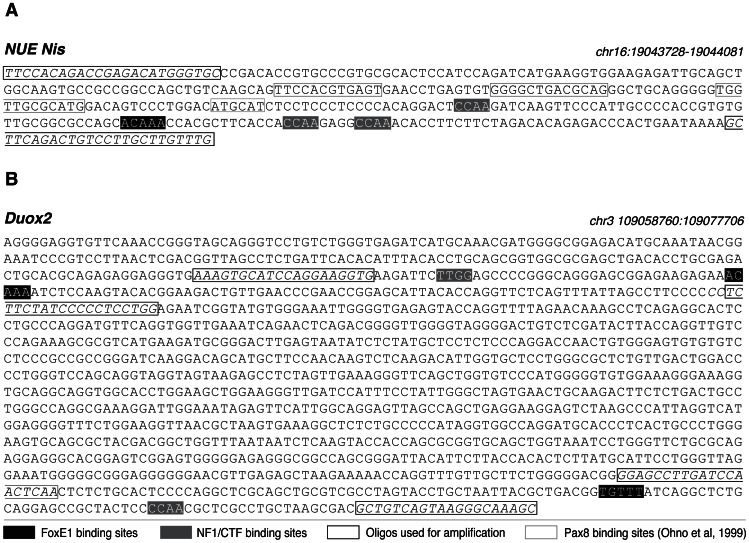
Putative FoxE1-NF1/CTF binding sites in the *Duox2* gene and in the NUE. Chromosomal location of putative FoxE1 and NF1/CTF binding motifs in the Nis upstream enhancer (panel A) and the *Duox2* gene (panel B). Oligos used and exons are represented in italics.

We also wanted to study in more detail the role of FoxE1 in Nis regulation (Fig. 2A). As we detected that Nis mRNA levels were even more strongly reduced than Tg mRNA levels, we tried to find FoxE1-NF1/CTF binding sequences in both the Nis proximal promoter (+/−1000 pb) and in the Nis upstream enhancer element (NUE ) localised between –2495 and –2264 bp [35]. A FoxE1-NF1/CTF binding sequence was detected in the NUE, close to a previously described Pax8 binding sequence [35] (Fig. 3A), but not in the proximal Nis promoter.

**Figure 4:**
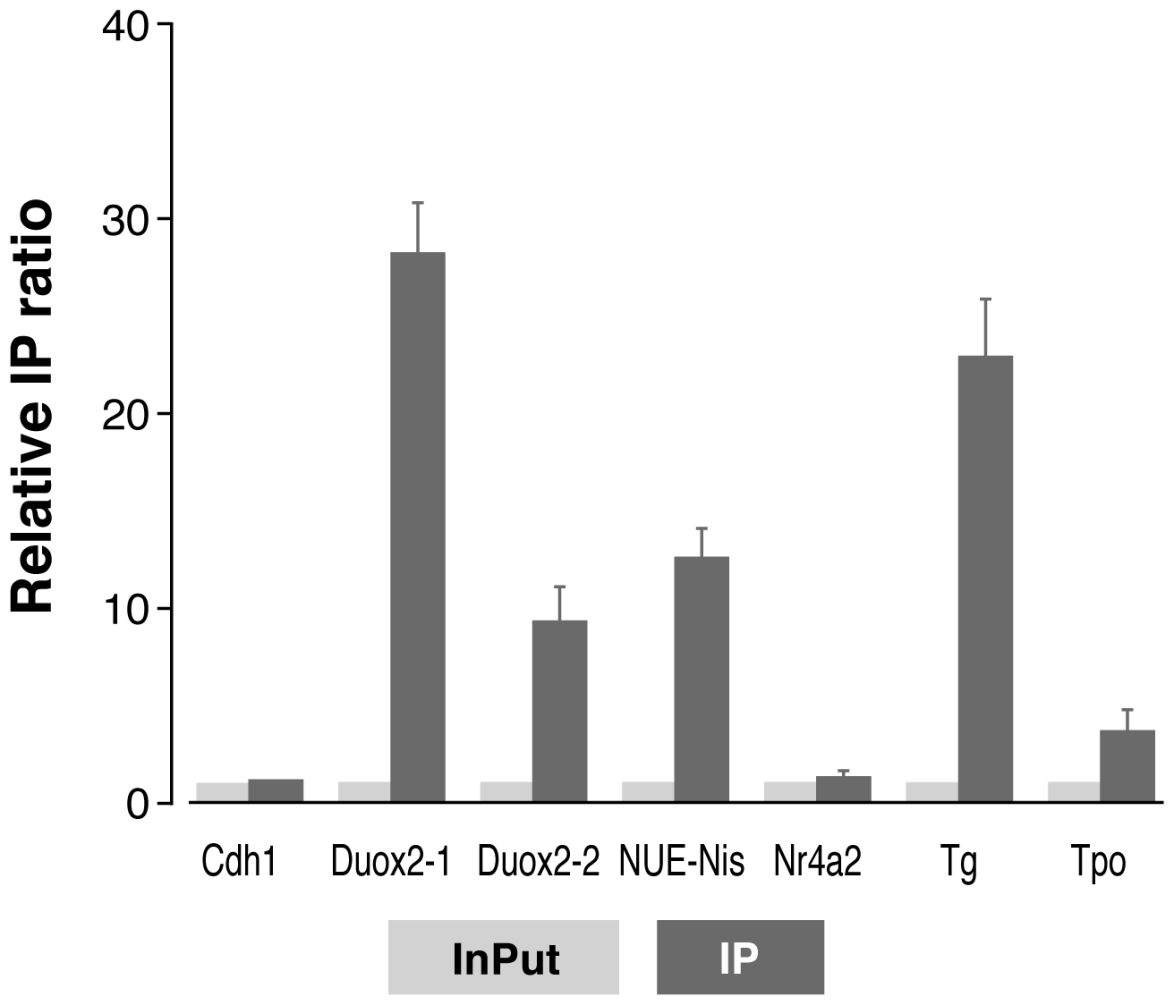
ChIP experiments for FoxE1 binding to selected genes. qPCR analysis of chromatin immunoprecipitation performed on PCCl3 cells with FoxE1 antibody. The enrichment of target sequences was calculated as the IP ratio (arbitrary units) relative to the negative control Afm, and normalized to their relative amplification in the input sample. Sequences from the Tg and Tpo promoters were used as positive controls. Two regulatory regions were analysed in the *Duox2* promoter (called Duox2-1 and Duox2-2). Results are mean ± SEM of two independent experiments, each performed in triplicate.

In order to analyse *in vivo* FoxE1 binding to *Duox2*, *Cdh1*, *Nr4a2* and *Nis* promoter sequences, we performed chromatin immunoprecipitation (ChIP) using a polyclonal antibody against FoxE1. We analysed immunoprecipitated DNA of two independent experiments using qPCR. Results of IP ratios normalized to the *Afm* negative control are shown in Fig. 4. The same results were obtained when we used *Gad1* as a negative control (data not shown). Previously described FoxE1 binding sequences in the *Tg* and *Tpo* promoters were used as positive controls.

The FoxE1 antibody precipitated FoxE1 target sequences in the *Tg* and *Tpo* promoters with relative immunoprecipitation (IP) ratios of 22.8 and 3.6, respectively. We found binding of FoxE1 to chromatin regions containing the *Nis* upstream enhancer element (relative IP ratio: 12.6). We also observed positive FoxE1 binding to two DNA sites located in the *Duox2* gene; one in the promoter sequence at −351 bp (relative IP ratio: 28.1) and the other in the third exon at +696 bp (relative IP ratio: 9.3) (Figs 3 and 4). We did not detect direct FoxE1 interactions with either *Cdh1* or *Nr4a2*.

In order to better understand whether FoxE1 and NF1/CTF are bound together on the *Nis* and *Duox2* promoter regions, we performed ChIP assays using a polyclonal antibody against NF1. Next, we also performed ReChIP experiments with FoxE and NF1 antibodies. We quantified immunoprecipitated DNA regions using qPCR. The previously described FoxE1 binding sequence in the *Tg* promoter was used as positive control.

**Figure 5:**
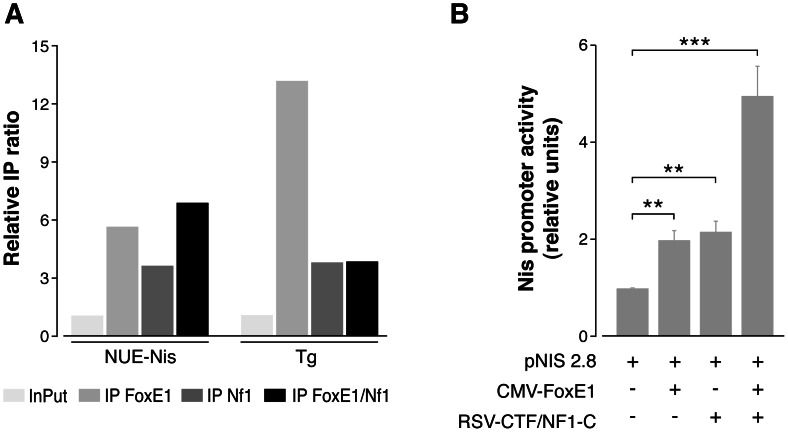
FoxE1 and NF1/CTF binding to and transcriptional activation of the NUE. A ReChIP assay was used to analyse simultaneous binding of FoxE1 and NF1/CTF proteins to the NUE (panel A). qPCR was done to analyse chromatin immunoprecipitates of PCCl3 cells using FoxE1 and NF1 antibodies. The enrichment of target sequences was calculated as the IP ratio (arbitrary units) relative to the negative control Afm, and normalized to their relative amplification in the input sample. A sequence from the Tg promoter was used as positive control. HeLa cells were transfected with 1 µg of a FoxE1 or NF1/CTF expression vector or the empty vector, and 1.5 µg and 0.1 µg of pNIS 2.8-Luc and CMV-*Renilla* constructs respectively (panel B). Forty-eight hours after transfection, cells were collected for the measurement of luciferase and *renilla* levels. Results are shown as the mean±SD of the luciferase levels relative to the non-regulated *renilla* levels of six independent experiments. (*): p<0.05; (**): p<0.01; (***): p<0.001; two tailed t-test.

In our experimental conditions, we did not observe *in vivo* NF1/CTF binding to either of the two putative FoxE1 binding sequences in *Duox2* (data not shown). Nevertheless, we detected positive binding of NF1/CTF to the NUE. Moreover, we observed simultaneous FoxE1 and NF1/CTF binding to the NUE region. Results of IP ratios normalised to the *Afm* negative control are shown in Fig. 5A. Similar results were obtained when *Gad1* was used as a negative control (data not shown).

As we wanted to study in depth the role of FoxE1 and NF1/CTF in Nis regulation, we cotransfected HeLa cells with FoxE1 and NF1/CTF expression vectors, together with a luciferase expression vector containing the NUE. Forty-eight hours later, cells were lysed and luciferase levels were measured. The results showed a significant 2-fold increase in the activity of NUE when FoxE1 or NF1/CTF were expressed individually in HeLa cells; thus, the effect was additive when both transcription factors were transfected together (Fig. 5B).

In summary, we present the first description of specific *in vivo* FoxE1 binding to regulatory regions of two relevant thyroid genes, *Nis* and *Duox2.* These FoxE1 core binding sequences are located close to a NF1/CTF transcription factor binding site. Moreover, we demonstrate that FoxE1 and NF1/CTF are simultaneously bound to the Nis upstream enhancer and that they cooperate in the regulation of NIS-promoter activity.

## Discussion

FoxE1 is a forkhead transcription factor essential for thyroid differentiation and function. In humans, *FOXE1* mutations cause the Bamforth–Lazarus syndrome (OMIM 241850) [9], [10]. In spite of this, FoxE1 expression levels seem to be unaltered in human tumours. As reflected in the Gene Expression Omnibus database, *FOXE1* variations have been associated with susceptibility to several types of cancer [11], [12], [13], including papillary thyroid cancer [14], [15], [16]. FoxE1 recognizes and binds to the *Tg* and *Tpo* promoters. However, little is known about other downstream targets of FoxE1 that could explain the phenotypes observed in humans carrying variations in *FoxE1*. Only two studies reporting novel genes regulated by FoxE1 have been published until now [22], [23], but both were done in a heterologous system overexpressing FoxE1. Our goal was to find new potential downstream mediators of FoxE1 function *in vivo*, but importantly, instead of a heterologous system we used a thyroid cell line. Results obtained by performing a genome-wide approach followed by chromatin immunoprecipitation analysis, led us to the identification of direct FoxE1 target genes in thyroid cells.

In this study, we found 54 putative novel FoxE1 target genes. We obtained a very restrictive list of significantly regulated genes, based on overlapping expression array data of two comparisons. This restrictive list did not allow us to obtain relevant information about main pathways affected by FoxE1 action. We identified and validated eleven genes regulated by FoxE1; four of them (*Adamts9*, *Cdh1*, *Duox2* and *S100a4*) showed an increased expression, whereas the remaining seven genes (*Casp4*, *Creld2*, *Dusp5*, *Etv5*, *Hsp5a*, *Nr4a2* and *Tm4sf1*) showed decreased mRNA levels when FoxE1 is silenced. Moreover, we have not ruled out that the other 43 statistically significant genes are true FoxE1 targets, and in future studies we will take advantage of these additional array results.

In order to identify genes directly regulated by FoxE1, we performed an *in silico* search of the FoxE1 binding motif in the promoter regions of 54 statistically significant genes. As the FoxE1 core binding sequence is very short, only five nucleotides, we were forced to perform a more restrictive search including the NF1/CTF binding sequence, as previously described for the *Tpo* promoter [21] and for other forkhead factors in the serum albumin gene promoter [34]. It has been proposed that the conserved interaction between NF1/CTF and forkhead proteins could reflect a general mechanism of action of both families of transcription factors. The NF1/CTF-binding site is masked inside the nucleosomal structure; binding of forkhead proteins to their cognate sites opens this structure and makes the NF1/CTF site accessible to exert its transactivation effect. The ability of the forkhead domain to induce DNA bending [36] would promote contact with NF1/CTF factors. It has been hypothesised that this interaction may play an important role in cell type-specific transcription and might be a widespread phenomenon [21].

*Duox2* codes for the enzyme that catalyzes H_2_O_2_ production in the thyroid gland [37], [38], [39]. *Duox2* expression is found in the thyrocyte more abundantly than in several other cell types [40]. In humans, mutations in the corresponding gene are associated with congenital hypothyroidism [41], [42]. *Duox2* regulation by the classical thyroid transcription factors Pax8 and Ttf1 has been studied in depth, however, two main studies reported controversial results [40], [43]. Nevertheless, until now there were no indications in the literature of a role of FoxE1 in Duox2 regulation.

In the present work we demonstrate that FoxE1 interacts *in vivo* with two DNA regions located in *Duox2*. We observed FoxE1 binding to the promoter region at −351 bp and to a second regulatory region located in exon 3 at +696 bp (Figs 3 and 4). However *Duox2* did not seem to be regulated by FoxE1 at the protein level, indicating the involvement of strong post-transcriptional regulatory mechanisms. Since *Duox2* mRNA expression increases when FoxE1 is silenced and since FoxE1 binds directly to two *Duox2* regulatory regions, we can hypothesise that FoxE1 is acting as a transcriptional repressor of *Duox2.*

The repressor function of FoxE1 has been previously reported. FoxE1 represses the transcriptional activity of Ttf1 and Pax8 during thyroid development through its C-terminal region, which contains an alanine-rich domain [3], [19]. FoxE1 also inhibits estrogen receptor α transactivation through this domain [44]. In line with our hypothesis, a direct repression function that may require both target DNA binding and dimerization domains [45] has been demonstrated for other forkhead family members such as FoxP.

FoxE1 binding domains in *Duox2* regulatory regions are localised at 26 and 27 bp from the NF1/CTF binding motif (Table 3). Nevertheless, in our experimental conditions, we were not able to detect direct *in vivo* binding of NF1/CTF to either of the two FoxE1 core sequences in *Duox2*. Thus, the exact FoxE1-mediated repression mechanism of *Duox2* expression remains to be elucidated.

Cdh1 belongs to a superfamily of glycoproteins that mediate calcium-dependent cell-cell adhesion. Cdh1 is highly expressed in the normal thyroid gland and its expression leads to aggregation of thyrocytes [46]. Moreover, thyroid-stimulating hormone (TSH) in dog and human thyroid cell cultures regulates *Cdh1* transcription [47], suggesting a relationship between *Cdh1* expression and thyroid differentiation. *FoxE1* expression itself is under TSH control [48]. Therefore it seems plausible that TSH, via FoxE1 and other players, might regulate expression levels of Cdh1. In fact, we observed an increased Cdh1 mRNA and protein expression when FoxE1 was silenced; nevertheless, we were not able to detect a direct interaction between FoxE1 and Cdh1.

Nr4a2 is a transcription factor that belongs to the steroid-thyroid hormone-retinoid receptor superfamily. In addition, Nr4a2 is a member of the NR4A subfamily of nuclear orphan receptors that function as ligand-independent early response genes involved in proliferation, apoptosis, and inflammation [49]. Nr4a2 cooperates with another member of the forkhead protein family, FoxA2, during dopamine neuron differentiation [50] and Nr4a2 overexpression enhances migration of mesenchymal stromal cells [49]. Our data reveals a downregulation of *Nr4a2* expression when FoxE1 is absent, but, in our experiments, FoxE1 did not directly bind to its promoter.

Further studies will be done to analyse in depth the apparently indirect regulation by FoxE1 of *Nr4a2* and *Cdh1* and its implication in cellular migration and/or thyroid differentiation.

Surprisingly, in the absence of FoxE, we observed both reduced *Nis* mRNA levels and reduced Nis protein levels (Fig. 2A and C). We found one FoxE1 core binding sequence and three NF1/CTF motifs in the NUE (Fig. 3A). NUE stimulates transcription in a thyroid-specific, cAMP-dependent manner and involves the most relevant aspect of *Nis* regulation. NUE contains Ttf1 binding sites that have no known effect on *Nis* transcription, Pax8 binding sites, and a degenerate cAMP responsive element sequence [35]. In their original study, Ohno *et al.* performed DNase I footprinting analysis of the NUE, but no footprints were detected when FoxE1 was used [35]. The first and foremost possibility that might explain the apparent discrepancy with our results is that Ohno *et al.* did *in vitro* experiments. In a later study, Li et al. suggested that FoxE1 participates in a human NIS repressor complex, together with PARP-1 [51]. Our study clearly demonstrates a direct *in vivo* interaction of FoxE1 with the NUE and a modification of *Nis* transcriptional activity in the absence of FoxE1. In addition, we observed direct NF1/CTF binding to the NUE, and we also demonstrated that FoxE1 and NF1/CTF are bound together on the NUE region. Binding of both transcription factors together resulted in an increased transcriptional activity of the NUE, confirming their cooperation in Nis regulation (Fig. 5).

Our work emphasizes the idea of a cross-talk of thyroid transcription factors during the process of transcriptional regulation, controlling key cellular processes for thyrocyte biology, with different roles of several players of this complex regulatory system that are still emerging.

On the one hand, a master thyroid gene, *Nis*, contains in its NUE binding sequences for Pax8, Ttf-1 and FoxE1-NF1/CTF that appear to be linked in a complex network of reciprocal regulatory interactions. On the other hand, another of the best candidate genes in our genome-wide study is the thyroid gene *Duox2*; thus, evidence is accumulating regarding the existence of a regulatory network in the thyroid involving FoxE1.

Finally, our study points out the importance of cooperating transcription factors and the usefulness of previously used tools, especially the *in silico* search of transcription factor binding sequences. Core sequences are necessary but not sufficient, and flanking sequences determine transcription factor binding specificity [23], [48]. The use of the FoxE1-NF1/CTF binding motif [21] led us to obtain a workable restrictive list of potential direct FoxE1 targets. Moreover, this opens a window to further investigating the cooperative function of FoxE1 and NF1/CTF in transcriptional regulation of our candidate genes, as we demonstrated to occur for Nis expression.

In search of potential downstream mediators of FoxE1 function, we identified *Nis* and *Duox2* as novel direct FoxE1 target genes. To our knowledge, this is the first evidence regarding the implication of *Nis* and *Duox2* in executing the transcriptional programme triggered by FoxE1. Both are pivotal proteins required for thyroid gland differentiation and function.

Although mainly acting as a regulator of the expression of the thyroid genes *Tg*, *Tpo*, *Nis* and *Duox2*, FoxE1 may also act in thyroid cells as a transcriptional regulator of many other candidate genes, some of which are suggested in the present study.

## Supporting Information

Table S1 **Oligonucleotides used for experimental validation of expression array data.**
(DOC)

Table S2 **Oligonucleotides used for ChIP analysis.**
(DOC)

Table S3 **Statistically significant probes (p<0.005) common to both comparisons (siFoxE1 PCCl3 vs siScrambl PCCl3, and siFoxE1 PCCl3 vs wt PCCl3).**
(DOC)

Dataset S1 **Microarray results for siFoxE1 PCCl3 vs siScramble PCCl3 comparison.**
(XLS)

Dataset S2 **Microarray results for siFoxE1 PCCl3 vs wt PCCl3 comparison.**
(XLS)
