# Supplementary material for: New Insights into FoxE1 Functions: Identification of Direct FoxE1 Targets in Thyroid Cells
Source: PLoS One. 2013 May 13;8(5):e62849. doi: 10.1371/journal.pone.0062849 (PMC3652843; doi:10.1371/journal.pone.0062849)
Supplement: Table S1 — Oligonucleotides used for experimental validation of expression array data. (DOC) [file pone.0062849.s001.doc]

**Table S1.**

| **Thyroid-specific Genes** | |  |  |  |  |
| --- | --- | --- | --- | --- | --- |
| **Primer Name** | **Array Status** | **Orientation** | **Sequence** |  |  |
| ***FoxE1*** | Downreg. | Forward | TCATCACCGAGCGCTTCCCGTT | | |
|  |  | Reverse | GCGGCTGCATCGTGCATGTA | | |
| ***Tpo*** | Downreg. | Forward | ATTGCACCAGATCATCACCA | | |
|  |  | Reverse | CCAAACCACCTTCCTGGATA | | |
| ***Tg*** | Downreg. | Forward | CAGGAAGGCTCTGCTTATGG | | |
|  |  | Reverse | CAGGAGCTTGGTCTGAGCTT | | |
| ***Nis*** | Downreg. | Forward | TGGACTTTGACCCTGATCCT | | |
|  |  | Reverse | ACAAGCAGCGGCATGTACT | | |
| ***Pax8*** |  | Forward | GGACAGTTGTCGACTGAGCA | | |
|  |  | Reverse | GAATGAGGATCTGCCACCAC | | |
| ***Ttf1*** |  | Forward | CAGGCCCGTCCCACCGAGAA | | |
|  |  | Reverse | TTGTGGTGGCCGCCCTTTCC | | |
| **Additional Genes** |  |  |  |  |  |
| **Primer Name** | **Array Status** | **Orientation** | **Sequence** |  |  |
| ***Adamts9*** | Upreg. | Forward | GAGCCGACGGGATGACTGCC | | |
|  |  | Reverse | CCCCAGCGGTGGCAAAAGGA | | |
| ***Casp4*** | Downreg. | Forward | TGGTGGTGAAAGAGGAGCTT | | |
|  |  | Reverse | GCACATCTGGAGTTGCTTCA | | |
| ***Cdh1*** | Upreg. | Forward | GTTTGCTCGGCGTTTGCCCG | | |
|  |  | Reverse | ACAAAGCCACGAGGAGACCTGC | | |
| ***Creld2*** | Downreg. | Forward | ACAAGTTCAACCAGGGGATG | | |
|  |  | Reverse | TTCCTGCTGCTCCAAGAGTT | | |
| ***Crip2*** | Upreg. | Forward | CCTCCAAGTGTCCCAAGTGT | | |
|  |  | Reverse | CGGAGGCTTCTCGTAGATGT | | |
| ***Duox2*** | Upreg. | Forward | CATGCTGCGCGACCACGACA | | |
|  |  | Reverse | TACAGCCGGGGAGAGGGAGC | | |
| ***Dusp5*** | Downreg. | Forward | CAGCCAGTGCGGAAAGCCCAT | | |
|  |  | Reverse | GCTAATGTCGGCGGTGTGGCT | | |
| ***Etv5*** | Downreg. | Forward | ACCACCCCCAAGGAACTACTCCA | | |
|  |  | Reverse | GGGCTGTGCAGCTCCCGTTT | | |
| ***Hspa5*** | Downreg. | Forward | TGCAGCAGGACATCAAGTTC | | |
|  |  | Reverse | CTGCATGGGTGACCTTCTTT | | |
| ***Nr4a2*** | Downreg. | Forward | ACGCGGCCTGTCAGCATTACG | | |
|  |  | Reverse | GGTAGACGACCTCTCCGGCCT | | |
| ***S100a4*** | Upreg. | Forward | GGGGAGAAGGACAGACGAA | | |
|  |  | Reverse | GCAGGACAGGAAGACACAG | | |
| ***Tm4sf1*** | Downreg. | Forward | CTACCGAGGGCCACCTCAGCC | | |
|  |  | Reverse | ACAAAAACGCCGGCAGGAGC | | |
| ***GusB*** | Reference | Forward | CATGACGAACCAGTCACCAC | | |
|  |  | Reverse | ACGGTCTGCTTCCCATACAC | | |
|  |  |  |  |  |  |
