# Supplementary material for: New Insights into FoxE1 Functions: Identification of Direct FoxE1 Targets in Thyroid Cells
Source: PLoS One. 2013 May 13;8(5):e62849. doi: 10.1371/journal.pone.0062849 (PMC3652843; doi:10.1371/journal.pone.0062849)
Supplement: Table S2 — Oligonucleotides used for ChIP analysis. (DOC) [file pone.0062849.s002.doc]

**Table S2.**

|  |  |  |
| --- | --- | --- |
| **Primer Name** | **Orientation** | **Sequence** |
| ***Cdh1*** | Forward | TGAAACAAGAGGATGGCTGA |
|  | Reverse | GGGAGGCATATACTCGCAAA |
| ***Duox2_1*** | Forward | AAAGTGCATCCAGGAAGGTG |
|  | Reverse | CAGGAGGGGGATAGAAGAGG |
| ***Duox2_2*** | Forward | GGAGCCTTGATCCAACTCAA |
|  | Reverse | GCTTTGCCCTTACTGACAGC |
| ***Nr4a2*** | Forward | CCCTGAGCTTCAACGAAGAC |
|  | Reverse | TGAAGGAAAGAAAGGGCTGA |
| ***NUE Nis*** | Forward | TTCTCTTTCCACAGACCGAGACATGGGTGC |
|  | Reverse | AGAGGCAAACAAGCAAGGACAGTCTGAAGC |
| ***Afm*** | Forward | ACCTGACAGCGAACACTGCTG |
|  | Reverse | TTCCAAACCGTTCCTGTGCAT |
| ***Gad1*** | Forward | GCCAGCACAGCCTTCCCGAAT |
|  | Reverse | GTGCGGTCCATGGGGACAACC |
| ***Tpo*** | Forward | AGCAAGGACACACAAGCACTT |
|  | Reverse | CTCCACTGAAGAAGCAGGCTG |
| ***Tg*** | Forward | TGTCCTGGAGTGGTCACCCTA |
|  | Reverse | CCAGTGTCCCATCTGAGT |
|  |  |  |
