# Supplementary material for: New Insights into FoxE1 Functions: Identification of Direct FoxE1 Targets in Thyroid Cells
Source: PLoS One. 2013 May 13;8(5):e62849. doi: 10.1371/journal.pone.0062849 (PMC3652843; doi:10.1371/journal.pone.0062849)
Supplement: Table S3 — Statistically significant probes (p<0.005) common to both comparisons (siFoxE1 PCCl3 vs siScrambl PCCl3, and siFoxE1 PCCl3 vs wt PCCl3). (DOC) [file pone.0062849.s003.doc]

**Table S3.**

| **Regulated genes in absence of FoxE1** | | |  |
| --- | --- | --- | --- |
| **Probe** | **Gene** | **Status** | **p-value** |
| A_43_P11489 | S100a4 | Upregulated | 1.91E-13 |
| A_44_P253208 | Adamts9 | Upregulated | 6.37E-12 |
| A_44_P142242 | Cdh1 | Upregulated | 3.66E-10 |
| A_42_P594793 | Prss8 | Upregulated | 7.41E-09 |
| A_64_P119916 | Duox2 | Upegulated | 4.49E-08 |
| A_64_P107892 | Folr1 | Upregulated | 9.92E-08 |
| A_64_P039576 | Elovl2 | Upregulated | 7.21E-07 |
| A_44_P220325 | Dynlrb2 | Upregulated | 1.03E-06 |
| A_64_P014183 | Crip | Upregulated | 1.49E-06 |
| A_44_P556319 | Fgf18 | Upregulated | 3.72E-06 |
| A_64_P108339 | Tmem140 | Upregulated | 4.16E-06 |
| A_44_P991532 | RT1-Da | Upregulated | 8.74E-06 |
| A_64_P067000 | Prima1 | Upregulated | 9.68E-06 |
| A_44_P449858 | Krt20 | Upregulated | 2.71E-05 |
| A_42_P453055 | Bcam | Upregulated | 1.18E-03 |
| A_42_P807866 | Ril | Upregulated | 1.24E-03 |
| A_64_P127604 | Slit1 | Upregulated | 2.68E-03 |
| A_42_P536741 | Derl3 | Downregulated | 1.54E-25 |
| A_43_P15271 | Ahcy | Downregulated | 1.99E-22 |
| A_44_P548559 | Derl3 | Downregulated | 4.36E-09 |
| A_44_P1054213 | Hspa5 | Downregulated | 4.89E-09 |
| A_44_P269930 | Mfsd2 | Downregulated | 4.89E-09 |
| A_43_P16457 | Creld2 | Downregulated | 4.96E-09 |
| A_44_P1045834 | Gmppb | Downregulated | 1.75E-06 |
| A_64_P019401 | Sdf2l1 | Downregulated | 1.75E-06 |
| A_64_P059495 | Ddit3 | Downregulated | 5.72E-06 |
| A_44_P171321 | Coq10b | Downregulated | 1.13E-05 |
| A_44_P415518 | Dnajc3 | Downregulated | 1.15E-05 |
| A_42_P664913 | Ankrd37 | Downregulated | 1.34E-05 |
| A_64_P074332 | Manf | Downregulated | 3.79E-05 |
| A_44_P635089 | Etv5 | Downregulated | 4.52E-05 |
| A_43_P15719 | Hsp90b1 | Downregulated | 1.15E-04 |
| A_64_P012586 | Ggct | Downregulated | 3.62E-04 |
| A_44_P127597 | Dnajb9 | Downregulated | 3.76E-04 |
| A_64_P064933 | Ggct | Downregulated | 3.85E-04 |
| A_64_P161630 | Amigo3 | Downregulated | 4.33E-04 |
| A_44_P1047467 | Igf2bp2 | Downregulated | 4.70E-04 |
| A_43_P11333 | Hyou1 | Downregulated | 8.85E-04 |
| A_44_P493956 | Pdia4 | Downregulated | 9.02E-04 |
| A_44_P517576 | Sel1l | Downregulated | 1.05E-03 |
| A_64_P078894 | Il23a | Downregulated | 1.08E-03 |
| A_64_P046798 | Tmem66 | Downregulated | 1.19E-03 |
| A_44_P1000561 | Atmin | Downregulated | 1.31E-03 |
| A_42_P457692 | Zfand2a | Downregulated | 1.39E-03 |
| A_44_P227616 | Engase | Downregulated | 1.45E-03 |
| A_42_P812008 | Nupr1 | Downregulated | 1.62E-03 |
| A_44_P1028549 | Casp4 | Downregulated | 1.72E-03 |
| A_64_P006873 | Ero1lb | Downregulated | 1.72E-03 |
| A_44_P139763 | Bet1 | Downregulated | 1.72E-03 |
| A_42_P554157 | Dnajb11 | Downregulated | 1.91E-03 |
| A_64_P012902 | Sdf2l1 | Downregulated | 1.93E-03 |
| A_42_P484738 | Ctgf | Downregulated | 2.00E-03 |
| A_64_P045804 | LOC365444 | Downregulated | 2.00E-03 |
| A_43_P17026 | Riok3 | Downregulated | 2.00E-03 |
| A_42_P739860 | Dusp5 | Downregulated | 2.53E-03 |
| A_43_P12023 | Nr4a2 | Downregulated | 2.87E-03 |
| A_43_P11152 | Tm4sf1 | Downregulated | 3.30E-03 |
| A_64_P017620 | Sec23b | Downregulated | 3.91E-03 |
